# The Zebrafish Information Network: major gene page and home page updates

**DOI:** 10.1093/nar/gkaa1010

**Published:** 2020-11-10

**Authors:** Douglas G Howe, Sridhar Ramachandran, Yvonne M Bradford, David Fashena, Sabrina Toro, Anne Eagle, Ken Frazer, Patrick Kalita, Prita Mani, Ryan Martin, Sierra Taylor Moxon, Holly Paddock, Christian Pich, Leyla Ruzicka, Kevin Schaper, Xiang Shao, Amy Singer, Ceri E Van Slyke, Monte Westerfield

**Affiliations:** The Institute of Neuroscience, University of Oregon, Eugene, OR 97403-1254, USA; The Institute of Neuroscience, University of Oregon, Eugene, OR 97403-1254, USA; The Institute of Neuroscience, University of Oregon, Eugene, OR 97403-1254, USA; The Institute of Neuroscience, University of Oregon, Eugene, OR 97403-1254, USA; The Institute of Neuroscience, University of Oregon, Eugene, OR 97403-1254, USA; The Institute of Neuroscience, University of Oregon, Eugene, OR 97403-1254, USA; The Institute of Neuroscience, University of Oregon, Eugene, OR 97403-1254, USA; The Institute of Neuroscience, University of Oregon, Eugene, OR 97403-1254, USA; The Institute of Neuroscience, University of Oregon, Eugene, OR 97403-1254, USA; The Institute of Neuroscience, University of Oregon, Eugene, OR 97403-1254, USA; The Institute of Neuroscience, University of Oregon, Eugene, OR 97403-1254, USA; The Institute of Neuroscience, University of Oregon, Eugene, OR 97403-1254, USA; The Institute of Neuroscience, University of Oregon, Eugene, OR 97403-1254, USA; The Institute of Neuroscience, University of Oregon, Eugene, OR 97403-1254, USA; The Institute of Neuroscience, University of Oregon, Eugene, OR 97403-1254, USA; The Institute of Neuroscience, University of Oregon, Eugene, OR 97403-1254, USA; The Institute of Neuroscience, University of Oregon, Eugene, OR 97403-1254, USA; The Institute of Neuroscience, University of Oregon, Eugene, OR 97403-1254, USA; The Institute of Neuroscience, University of Oregon, Eugene, OR 97403-1254, USA

## Abstract

The Zebrafish Information Network (ZFIN) (https://zfin.org/) is the database for the model organism, zebrafish (*Danio rerio*). ZFIN expertly curates, organizes, and provides a wide array of zebrafish genetic and genomic data, including genes, alleles, transgenic lines, gene expression, gene function, mutant phenotypes, orthology, human disease models, gene and mutant nomenclature, and reagents. New features at ZFIN include major updates to the home page and the gene page, the two most used pages at ZFIN. Data including disease models, phenotypes, expression, mutants and gene function continue to be contributed to The Alliance of Genome Resources for integration with similar data from other model organisms.

## INTRODUCTION

The Zebrafish Information Network (ZFIN) (https://zfin.org/) is the central repository for zebrafish (*Danio rerio*) genetic and genomic data. ZFIN collects, organizes and makes available a wide range of data on zebrafish, including genes, gene function, sequences, mutants, transgenic lines, human disease models, expression, phenotype, orthology, sequence targeting reagents and antibodies. Data at ZFIN can be accessed through ZFIN search interfaces, download files, and ZebrafishMine (zebrafishmine.org), a data mining resource (1,2). Community services include nomenclature support for genes and alleles, pages for researchers, laboratories and companies, and a wiki for researchers to share antibody and protocol information. Here we describe usability updates and new features on two of the most used pages at ZFIN: the zfin.org home page and the gene page.

## NEW FEATURES

The zfin.org home page and gene page are the two most frequently visited pages at ZFIN, accounting for 7% and 16% of total pageviews in 2019 respectively. Recent annual surveys of the zebrafish research community included questions about how these two pages could be improved to provide increased functionality. Based on this input, the ZFIN home page and gene page have been completely redesigned to address the most significant needs of the zebrafish research community.

### New home page

**Figure 1.**
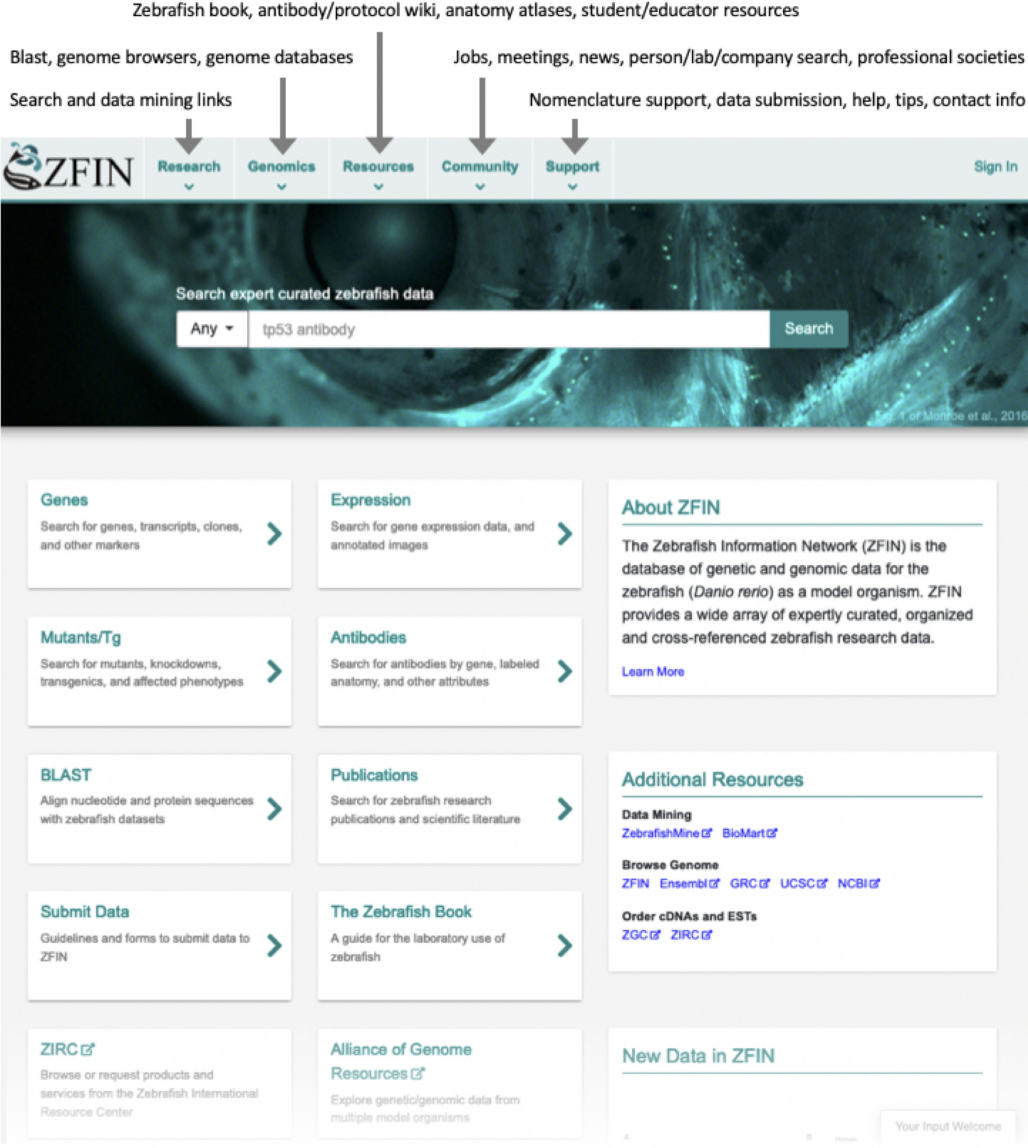
The new zfin.org home page. The new zfin.org home page showing site-wide menu choices (top), search box (middle) and link buttons to the most used features (bottom left). The lower portion of the home page is not shown, including sections for ‘New Data in ZFIN’, as well as jobs, meetings and news posts.

Annual community surveys indicated that the most important home page features were to maintain prominent search capability, improve organization and usability of links, and continue to provide listings of community news, jobs and meetings. These features became the central focus around which the new home page was designed (Figure 1). Links were organized into a set of drop-down menus located at the top of every ZFIN page and the single box search was made more prominent. The default is to search over all the data categories in ZFIN. Specific data types can be searched by selecting one from the pulldown menu to the left of the search box. Search categories include gene/transcript, expression, phenotype, human disease, fish, reporter line, mutation/Tg, construct, sequence targeting reagent, antibody, marker/clone, figure, anatomy/GO, community and publication.

Easy access to the most frequently used resources was supported with an array of 10 prominent buttons. These include searches for genes, expression, mutants, antibodies, and publications. Links to Blast, The Zebrafish Book, data submission guidelines, The Zebrafish International Resource Center (ZIRC) and The Alliance of Genome Resources (The Alliance; alliancegenome.org) (3) are also included. A concise description of what ZFIN is and a small set of mostly external frequently used additional resources were also added.

Other new features on the home page, not shown in Figure 1, include recently posted jobs, news, and meetings which are linked to the ZFIN wiki for details and complete listings and an image carousel to highlight data recently added to ZFIN. The carousel is automatically populated daily with ten images showing curated phenotype or expression data (*in situ* hybridization or immunohistochemistry) from papers published in the previous twelve months.

### New gene page

The gene page is the most frequently visited page on zfin.org. Recent community surveys identified changes to improve navigation, usability and delivery of key information for several data types shown on the gene page.

#### Navigation and section visibility

Both the volume of data and number of data types shown on gene pages have increased over time, making it more difficult for users to identify and locate sections for each data type. To address that, data sections and data tables were made more distinct and a navigation panel was added to the left page margin, allowing one click access to any specific data type from anywhere on the gene page.

#### Expression and phenotype sections

Community surveys indicated that the gene expression and phenotype data sections required the same new features: direct access to full annotations, associated images, and the publications from which they originated. Because expression and phenotype data share a similar annotation structure (location, developmental stage, image, citation) and needed the same updates, the two sections were redesigned to use the same new user interface in each section. The two sections are thus considered together here.

**Figure 2.**
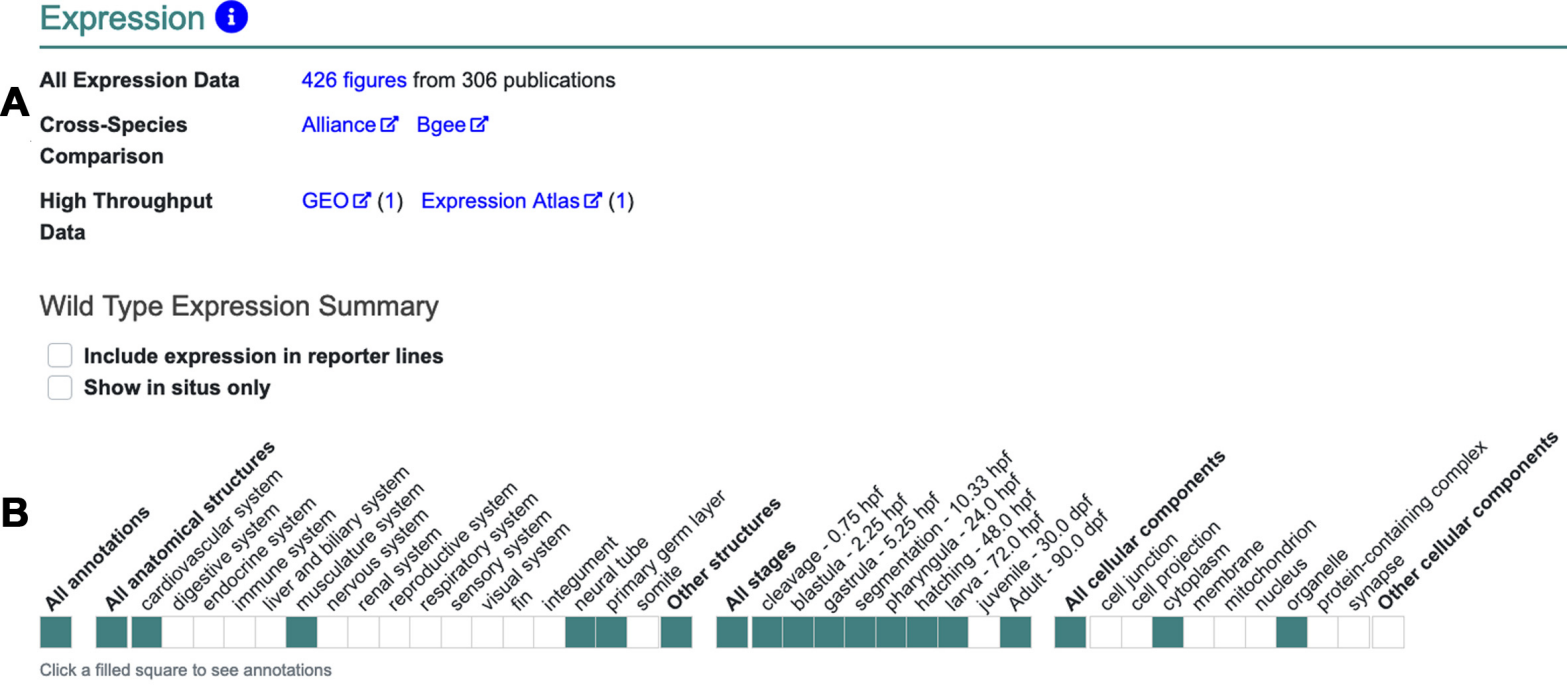
The default view of the new expression section on the *myl7* gene page. (**A**) Links to all expression data in ZFIN for the gene, cross species-comparisons for expression of the gene at The Alliance or Bgee, and high throughput expression data sets at GEO or Expression Atlas. (**B**) The wild type expression summary ribbon. Colored blocks indicate where annotations have been made.

For each data type a summary overview is provided. The summary includes how many figures and publications have been curated, links to additional resources, and a new summary ribbon. The summary ribbon provides a high-level overview of which anatomical systems and GO processes have annotated data. The default view for the expression section includes links to all expression data in ZFIN for the gene, a cross species comparative expression tool at the Alliance and Bgee (bgee.org) (4), and high throughput expression data at GEO (5) and Expression Atlas (6) (Figure 2A). For the phenotype section, the default view includes a link to all the phenotype data in ZFIN that involves the gene and a link to phenotype data at The Alliance. Both the expression and phenotype sections also display a summary ribbon to provide an overview of the data (Figure 2B).

The expression summary ribbon includes three sections to summarize wild-type expression for the gene using terms from the following ontologies: anatomical systems/structures (Zebrafish Anatomy ontology; ZFA), developmental stages (Zebrafish Stages ontology; ZFS), and cellular components (Gene Ontology; GO) providing a high-level summary of where and when a gene is normally expressed. Options are also provided to include expression in reporter lines, which are excluded by default, and to include only in situ hybridization data from publications and direct data submissions such as the Thisse datasets (7,8). The phenotype summary ribbon includes five sections to summarize the phenotype for the gene using terms from the following ontologies: anatomical structures (ZFA), developmental stages (ZFS), molecular functions (GO), biological processes (GO) and cellular components (GO). For both expression and phenotype, the terms used in the summary ribbon cover the broadest set of terms based on existing annotations in ZFIN.

**Figure 3.**
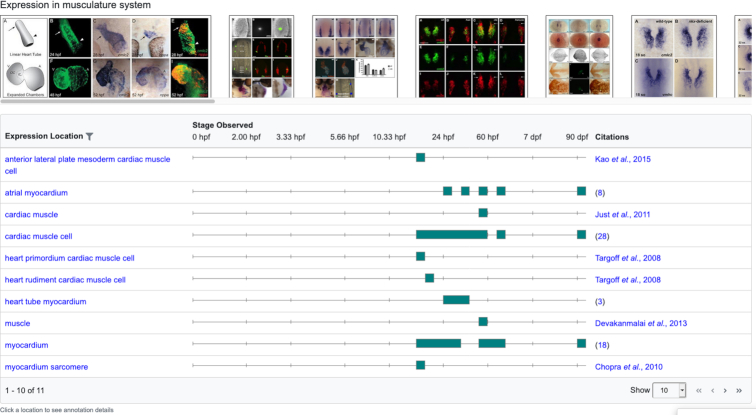
Expression image gallery and annotation summary table for *myl7* in the musculature system. Clicking on colored blocks in summary ribbons opens a new view including an image gallery and annotation summary table. Hovering over one of the colored developmental stage blocks provides a listing of specific stages included in that block.

The summary ribbons also provide access to the underlying annotations. Clicking a filled box in a summary ribbon opens an image gallery and annotation table. For example, clicking on the colored block for ‘musculature system’ in the *myl7* expression ribbon shown in Figure 2 produces images and a data table with all the annotations available at ZFIN for expression of *myl7* in any part of the musculature system (Figure 3). The image set contains any image available at ZFIN that is associated with an annotation in the annotation summary table.

Clicking on an image provides a larger view of the image with associated metadata and the caption as well as a link to the full figure page. Not all expression or phenotype annotations have images due to copyright restrictions, so in some cases no images may be available. For expression data, the data table produced after clicking a shaded box in the summary ribbon includes a distinct listing of the locations of wild-type expression. The display also includes developmental stages when expression was observed shown as teal colored blocks on a developmental timeline, and citations for those data. For phenotype data, the data table produced after clicking a box in the summary ribbon includes a unique list of full phenotype statements, associated developmental stages at which the phenotype was observed, and the citations for those data. In most cases, this view of the data will provide enough detail for researchers to take the next steps to explore the data. If specific annotation details are of interest, users can click on the expression location or phenotype statement to get the full annotation details table for that location or phenotype and reduce the image set to just those images associated with that location or phenotype (not shown). For expression, the full annotation details include the fish (genotype+knockdown reagents), experimental conditions, assay, stage, figure and citation for expression in that specific location. For phenotypes, the full annotation details include the fish, experimental conditions, stage, figure and citation for that specific phenotype. A video tutorial illustrating how these new features work is available on the ZFIN YouTube channel at https://youtu.be/XhcxEY4yfqE.

#### Gene ontology section

Gene function is described via Gene Ontology (GO) annotations which are created by associating a gene and gene product with a GO term. These GO terms can refer to the molecular function (MF) that the gene product enables, the biological process (BP) in which the gene product is involved, and the cellular component (CC) in which the gene product performs its function (9).

**Figure 4.**
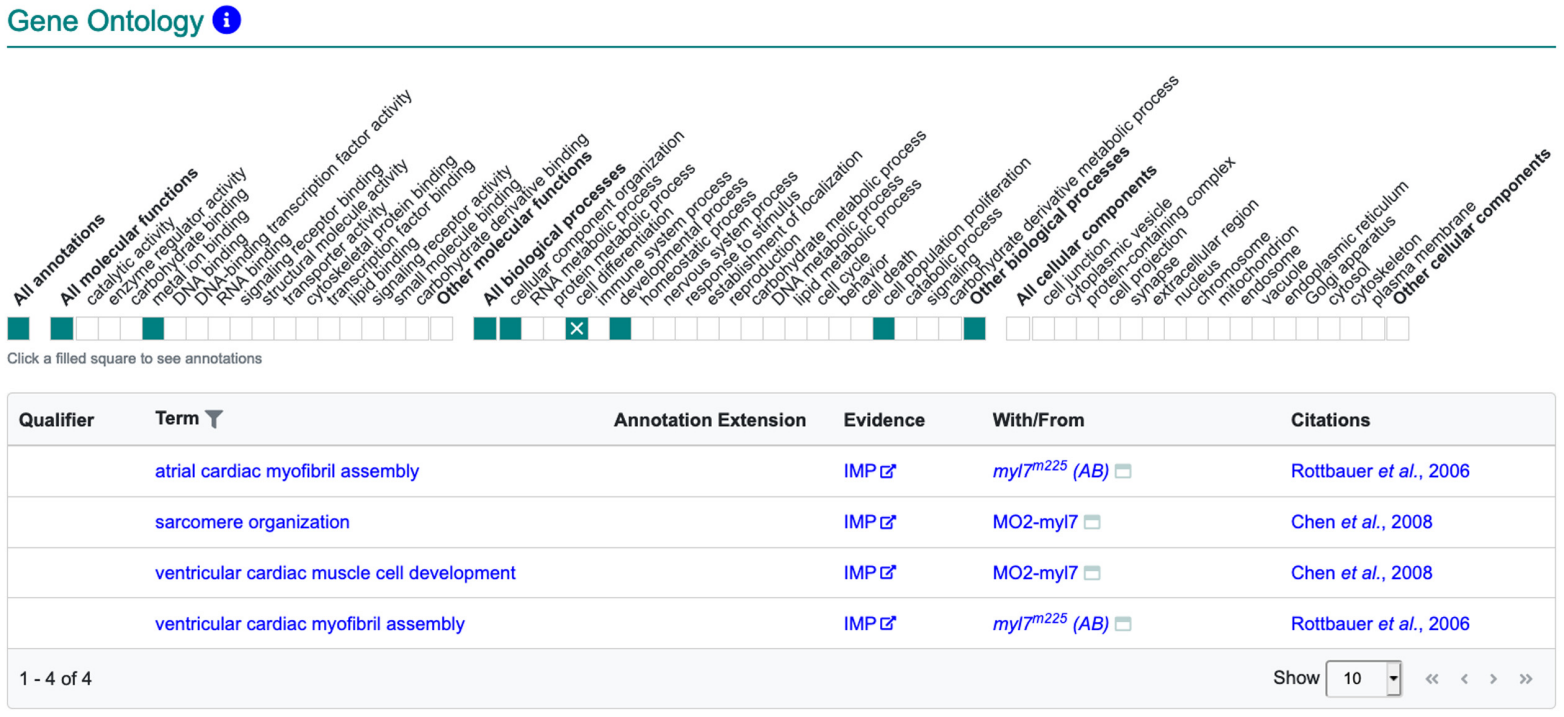
The Gene Ontology section for *myl7*. The default view shows the summary ribbon. Clicking on a colored block in the ribbon opens the data table. Shown here is the display for the *myl7* gene including the annotation table after clicking on the ‘cell differentiation’ block in the biological process portion of the GO ribbon.

The GO section on the gene page utilizes the summary ribbon display to provide high-level summary of gene function. Clicking on a filled block in the ribbon produces the associated GO annotations including the annotated GO term, evidence code, with/from field and citation (Figure 4).

Terms used in the GO summary ribbon represent high-level, broader terms for each of the aspects (molecular function, biological process and cellular component) of the underlying annotations. These high-level terms were chosen in collaboration with the GO Consortium and the Alliance of Genome Resources based on annotation coverage for model species represented at the Alliance.

#### Additional gene page updates

**Figure 5.**
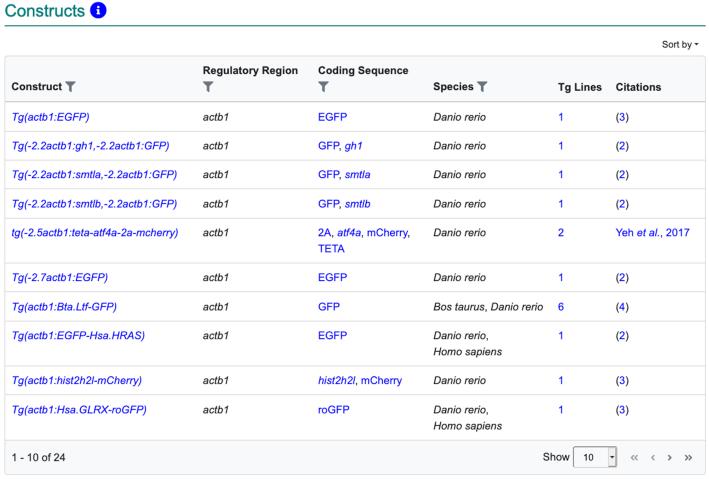
The new constructs table for the *actb1* gene. The data in the constructs table help researchers decide which construct is of interest to them without having to visit each individual construct page.

All sections of the gene page were updated in the new page design. Prior versions of the gene page provided simple lists of relevant reagents such as antibodies or knockdown reagents. It was necessary to visit individual pages for each of these reagents to determine their relevance for the task at hand. One major upgrade was to convert these lists to data tables on the gene page including additional data (Figure 5). The additional data were chosen to provide sufficient information to decide whether a specific record is of further interest without having to visit each record individually. New data tables are now included for six types of data on the gene page: mutations and sequence targeting reagents, constructs, sequence information, antibodies and citations sections.

To enhance data exploration, filtering, sorting, and pagination have been included in the tables to support viewing the data in multiple ways (Figure 5). Row sorting functionality has been integrated in the data table and supports multiple data sorting options (Figure 5). Filtering is also available in the column headers for many of these tables. The filter box gives the ability to search for a text string and return a filtered set of matching results. The filter box is either directly displayed in the section (as seen in the citations section) or can be accessed by clicking on the ‘funnel’ icon in the column header of the table (as seen in Figure 5). Filtering and sorting data tables are demonstrated in a tutorial video on the ZFIN YouTube channel at https://youtu.be/7UvLrWUDs4. To enhance readability, tables include an option to show 10, 25 or 100 rows. Pagination controls are also provided to access all records in the table without leaving the gene page.

The new layout used for the gene page has also been implemented for additional marker pages in ZFIN including transcript, antibody, STR, clone and other marker pages to provide the same user interface across all marker pages. These new page layout and UI features will also be used in other pages at ZFIN as those pages are updated.

## Improved mutant information

The precise genome location and sequence of alleles/variants are crucial to compare phenotype data and display location, protein effects predictions, cross-species comparison and translational research applications. Genome browsers (such as JBrowse (10)) and tools to predict the effects of sequence alterations on transcripts and proteins (such as the Ensembl Variant Effect Predictor (VEP) (11)) require this genomic coordinate information. Precise genomic coordinate locations must include the genome build, chromosome, nucleotide base position start and end, and nucleotide change (reported for the genomic DNA plus strand) to locate and identify sequence variants properly. This detailed information is rarely reported in publications and therefore was often unavailable for alleles/variants curated at ZFIN from publications.

ZFIN dedicated effort to manually determine and record the detailed variant information for the alleles/variants available at ZFIN. This was possible only when enough information (such as mutation sequencing data) was available in the publication or by direct communication with authors. As of 6 June 2020, we were able to add variant information for 50% (2250 out of 4469 total) of manually curated alleles/variants (point mutation, deletion, insertion, MNV, and delins types), leading to a total of 94% (39 088 out of 41 514 total) of alleles available at ZFIN which have known genomic information.

Precise genomic coordinate locations allowed us to display more variants in GBrowse at ZFIN, offering a more complete view of available alleles/variants within a gene or a genomic region. It also allowed us to retrieve and display the genomic sequences flanking the variant/mutation, which is a feature that had been requested by users. Five hundred bases of positive strand flanking sequence 5’ and 3’ of known variant locations were fetched from the GRCz11 genome sequence at Ensembl using a Java API for high-throughput sequencing data (HTS) formats. These data are updated weekly for newly added mutant locations. Having precise variant information also allows for providing this information to the Alliance where it is being displayed in JBrowse and the Alliance Sequence Viewer, and will ultimately be used for cross-species comparison. The submitted variants are also run through the Alliance VEP pipeline which predicts the effect of these variants on transcripts.

## ZFIN AND THE ALLIANCE

ZFIN is one of the founding members of the Alliance of Genome Resources, which originated in 2016. The Alliance has the mission to develop and maintain sustainable genome information resources that facilitate the use of diverse model organisms in understanding the genetic and genomic basis of human biology, health and disease. Since origination, data available at the Alliance has consistently grown. As of Alliance software and data release 3.1 (1 July 2020) ZFIN contributes the following data types: genome data, wild type expression, phenotypes, alleles/variants, disease models, orthology and gene function (gene ontology), as well as a limited set of curated metadata for RNAseq datasets. These data are in turn used to show the zebrafish genome, genes, and alleles/variants in the Alliance genome browser and to create gene and other pages at the Alliance. Much of the data included at the Alliance, for all the included model organisms, are now available via Alliance download files (https://www.alliancegenome.org/downloads). These data are also used at the Alliance to create automated gene descriptions for each gene (12). These descriptions are in turn added back to ZFIN and shown near the top of each gene page. In addition to sharing data, software can also be reused. The new summary ribbons in ZFIN were created using the ribbon code originally created by the GO consortium and used at the Alliance.

## DATA AVAILABILITY

The new home page and gene page were created with a better awareness of modern responsive design best practices. This new approach provides significantly improved support for mobile devices. The layout of links and sections on the new home page automatically resizes and reorganizes to be effective on a variety of screen sizes. The new site-wide header menu behaves similarly, with a menu system that collapses on small screens. The tables and ribbons on the new gene page were designed from the start with horizontal scrolling capability to work on any screen size. Work will continue to improve mobile access for all pages in ZFIN.

Data continue to be available via tab-delimited download files (https://zfin.org/downloads) as well as via the ZFIN InterMine (13) instance, ZebrafishMine, (http://ZebrafishMine.org; https://github.com/ZebrafishMine/intermine), which also serves ZFIN data via web services.

## TECHNICAL IMPLEMENTATION

The ZFIN web architecture is written primarily in Java using the Spring Framework and served via JSP in Apache/Tomcat. In addition, ZFIN implements new user interface components in React. Groovy, SQL, and Perl are used primarily to process and load data in bulk. Hibernate serves as the object-relational mapping library from Java to a PostgreSQL relational database. ZFIN uses both Solr and Java/Spring to facilitate search interfaces. Data from papers are entered via a web-based curation interface primarily written in GWT with some interfaces implemented in AngularJS. The community wiki is powered by Atlassian Confluence software (http://www.altassian.com/software/confluence/). A detailed and browsable view of the current ZFIN data model can be found at http://zfin.org/schemaSpy/index.html.
